# Interaction between overconfidence effects and training formats in nurses’ education in hand hygiene

**DOI:** 10.1186/s12912-024-02020-w

**Published:** 2024-07-02

**Authors:** Julia Seidel-Fischer, Milena Trifunovic-Koenig, Bianka Gerber, Baerbel Otto, Michael Bentele, Martin R. Fischer, Stefan Bushuven

**Affiliations:** 1 Academy for Health Care Professionals, Health Care Association District of Constance, Constance, Germany; 2 Training Center for Emergency Medicine (NOTIS e.V), 78224 Engen, Germany; 3 Wiesbaden Institute for Healthcare Economics and Patient Safety, Wiesbaden Business School, Rhein-Main University of Applied Sciences, Wiesbaden, Germany; 4 Institute for Anesthesiology, Intensive Care, Emergency Medicine and Pain Therapy, Hegau-Bodensee Hospital, Singen, Germany; 5 Institute of Medical Education, LMU University Hospital, LMU Munich, Munich, Germany; 6 Department of Anesthesiology and Critical Care, Medical Center – University of Freiburg, Faculty of Medicine, University of Freiburg, Freiburg, Germany; 7 Institute of Laboratory Medicine, LMU University Hospital, LMU Munich, Munich, Germany

**Keywords:** Hand hygiene, Hand disinfection, Learning format, Metacognition, ICAP-model, Nursing, Nursing students, Education, Overconfidence

## Abstract

**Background:**

Undergraduate training in hand hygiene is a keystone of infection control. Several studies have shown overconfidence effects in hand hygiene practices, which can impair metacognition. We hypothesized that overconfidence might be prevalent in the early education stages of nursing students and that these effects could be reduced through frequent interactive learning formats, such as learning groups.

**Methods:**

We conducted a multicenter cross-sectional questionnaire with 196 German nursing students, including general, surgical, and anesthetic nursing specializations.

**Results:**

Overconfidence was observed in nursing students across all specialties and years of education. The cluster analyses showed three different types of learners: two characterized by overconfidence and one demonstrating justifiable confidence. Furthermore, the moderation analysis indicated that providing feedback and promoting metacognition regarding students’ learning achievements could mitigate overplacement, particularly through the frequent implementation of interactive teaching formats.

**Discussion:**

Despite some limitations, these findings highlight the prevalence of overconfidence effects in nursing students, the presence of different learning profiles, and the importance of incorporating feedback within interactive learning formats concerning hand hygiene. Accordingly, educators need to be trained and supervised to deliver these learning formats and provide feedback to students effectively.

**Supplementary Information:**

The online version contains supplementary material available at 10.1186/s12912-024-02020-w.

## Introduction

### Background

Hand hygiene is the cornerstone of infection prevention and is widely acknowledged as the most efficient and cost-effective intervention in reducing hospital-acquired infections. Furthermore, hand hygiene represents a substantial component of patient safety [1]. Hence, training programs in hand hygiene are widely integrated into the curricula of physicians, nurses, and other health care professionals (HCPs). Beyond the procedural aspect, the competency to “speak up” is vital in fostering a secure environment for both HCPs and patients, particularly in instances where hand hygiene is omitted or performed incorrectly [2]. Several studies have demonstrated potential for improvement of both hand hygiene practices and speaking up [2–5].

Since 2018, our workings group and other authors have identified overconfidence effects (OCE) in hand hygiene practices and feedback reception skills among HCPs across different professions and workplace settings within local and national populations [6–10]. OCE [11] are also incorrectly referred to as “flawed self-assessment” or the Dunning-Kruger-effect in the popular science [12]. OCE are considered inherent to human nature and manifest in various social contexts, such as economy [13] criminollogy [14], and education [15]. While many scientists interpret the Dunning-Kruger-effect as statistical bias [16], only one study has described detectable physiological reactions associated with the Dunning-Kruger-effect [17]. In contrast to the Dunning-Kruger-effect OCE is considered a genuine phenomenon and can be categorized into three sub-effects: the overestimation effect, which is absolute (“I am better than objectively measured”), overplacement or better-than-average, a relative effect (“I am better than average”), and overprecision, the strong conviction of accuracy in self-assessment [11]. Collectively, these effects not only influence subjective self-assessment but also impede self-reflection and metacognition [18]. Given that traditional learning formats often fail to reduce OCE [19] interactive or constructive learning approaches are deemed more suitable for addressing OCE and stimulating metacognition (the “thinking about thinking”). Metacognition in education, initially conceptualized as the capacity to observe, evaluate, and strategize one’s own learning process [20], hinges on self-reflection. It consists of two key components: metacognitive knowledge and metacognitive control [21]. Knowledge includes understanding information processing and learning tasks, while control regulates cognitive processes for effective task completion [22]. Feedback plays a crucial role in promoting metacognition by enhancing self-reflection and awareness of one’s learning process. When individuals receive feedback on their performance, they are usually prompted to evaluate their understanding, identify areas for improvement, and adjust their strategies accordingly. This reflective process enhances metacognitive skills, such as monitoring and regulating cognitive processes, ultimately leading to more effective learning outcomes [23, 24]. To provide a structured approach to understanding various learning formats in medical education the Interactive-Constructive-Active-Passive (ICAP) framework offers a valuable lens. This framework delineates between four distinct formats: passive (e.g. passive absorption of the learning content), active (e.g. presentations with active components like writing, answering questions), constructive (e.g. reflection, written homework, development of mind maps and completing tasks in the classroom), and interactive learning formats (e.g. learning in interacting groups). The model offers a detailed taxonomy of cognitive engagement modes of the learners crafted by Chi and Wylie (2014) [25]. This model enables educators to discern how students interact with instructional tasks, ranging from passive listening to active knowledge co-construction. By elucidating these behaviors, the framework facilitates the design of more effective pedagogical approaches within medical education contexts [25, 26].

In addition to findings of two prior studies on hand hygiene conducted by Lengerke and colleagues [10], our previous research involved a cohort of more than 1000 medical students from various German universities. Our study revealed that these effects are consistent across all universities and manifest from the early stages of medical education. Furthermore, we could show that medical students assessed themselves to have superior hand hygiene practices compared to postgraduate supervisors and nurses [27]. This phenomenon can be attributed to the clinical tribalism, wherein the sense of optimal hand hygiene compliance extends beyond individual behavior to encompass group identification (“we against them”), akin to an “in-group bias” [28].

These findings raise the question of to what extent OCE may impair the quality of hand hygiene training of undergraduate and postgraduate HCPs, such as nurses or specialized medical assistants. They partially share training, fostering interprofessional education (learning together and from each other), rather than solely multi-professional (learning together) education [29].

During the study period in Germany, three main educational courses were available for non-academic HCPs, each spanning three years: nurses (“Gesundheits- und Krankenpfleger: in”, GKP) including general, pediatric, and geriatric nurses; anesthesia technical assistants (“Anästhesietechnische: r Assistent: in”, ATA); and surgical assistants (“Operationstechnischer: r Assistent: in”, OTA). Additionally, qualified nurses had the opportunity to pursue a specialized postgraduate training in anesthesiology and critical care (“Weiterbildung Intensivpflege und Anästhesie”). The nursing and ATA/OTA training curriculum in Germany adheres to government guidelines. Theoretical lessons take place at schools for health professions authorized by the state, while practical training takes place within the different specialized hospital departments. Certain courses currently provide the opportunity to complete the training at universities. The schools develop a curriculum based on the recommendations of the framework curriculum. Hygiene and infection prevention are important aspects of the training content. During learning sessions, topics such as hygienic hand disinfection, infection sources, transmission routes, nosocomial infections, cleaning, disinfection and sterilization processes, and the legal basis for infection protection are comprehensively discussed. However, there is no predetermined number of teaching hours allocated to hygiene education.

When designing lessons, the focus lies on attaining professional competence, and the learning sessions should be designed to be action-oriented and methodically varied. However, it remains challenging to align teaching with the competence goals. The question arises as to whether classic teaching methods outweigh action-oriented teaching, thereby potentially failing to adequately facilitate competence acquisition. With regard to professional competence, the teaching design should be counter-characterized by individual appropriation of the learning content, discursive discussion, independent control and reflection of the learning process, and learning actions within a protected framework [30].

### Rationale

The objective of this study was to clarify whether OCEs in hand hygiene, speaking up, and feedback reception skill manifest among German GKP, ATA, and OTA, similar to findings observed in preceding studies involving postgraduate professionals and medical students.

We hypothesized that:

1. Overplacement (better-than-average) effects can be detected in undergraduate nursing students (H1).
2. Overestimation effects are present in undergraduate nursing students (H2).
3. Furthermore, we hypothesized that teachers, who stimulate self-reflective learning by providing students with feedback and encouraging them to reflect on their current stage of knowledge and learning achievements (“metacognition”), can provoke a significant reduction of overplacement.

## Methods

### Study design and setting

Prior to the COVID-19 pandemic, we conducted a cross-sectional online questionnaire at 25 nursing schools in Germany that offer the GKP, ATA and OTA programs from July to September 2019. The study took place following the investigation of medical students [27]. The study protocol was approved by the ethics committee of the physician association Baden Wurttemberg (Landesaerztekammer Baden-Wuerttemberg). We used the questionnaire platform “umfrageonline.com” by Enuvo GmbH, Zurich, Switzerland. The English version of the questionnaire is provided in Supplement A.

### Participants

In total, approximately 3000 students (1800 GKP, 750 ATA and 750 OTA) across 25 medical schools were contacted, resulting in 196 responses. The inclusion criteria necessitated the current registration as a GKP, ATA, or OTA student. In Germany, a contractual agreement between students and the school is obligatory due to the practical nature of the work involved. Students have the option to commence nursing school and formalize their commitment after completing the 10th grade at the age of 16. Alternatively, if they leave school after the 9th grade at the age of 15, they must undergo training as a nursing assistant, albeit without registration as GKP, ATA, or OTA students and after that with 16 begin their nursing training. Consequently, participants had to be at least 16 years old to meet the criteria to be registered as nursing students and to take part in this study [31]. There were no other exclusion criteria. Participants voluntarily took part in the study and received information through an invitation letter from their respective faculties along with a pre-briefing provided within the questionnaire.

### Variables

General data included the educational progress in years, gender, age, previous vocational education, year of exposure to the first training in hand hygiene and skills lab experience. These variables were followed by the SATIS-4D questionnaire [27] using a 5-point Likert scale (five (completely agree), four (moderately agree), three (partially agree), two (agree somewhat), one (completely disagree)).

### Bias

The bias for convenience sampling was addressed according to Ball [32], as we mainly expected selection bias.

### Study size

We aimed to reach at least 100 nursing students. This objective could be achieved by repetitive communication and letters to faculties of nursing schools and nationwide organizations and associations.

### Quantitative variables relevant to the current study

*Self-assessment.* We used a six-item instrument (Cronbach’s alpha (α) = 0.67, McDonald’s Omega (ω) = 0.67) to measure the self-assessment of proficiencies in infection prevention and control (IPC). The scale measures the following aspects of the proficiencies: three items address the situational application of the factual knowledge in hygienic hand disinfection (e.g., “I conduct hand hygiene if indicated in a situation”), one item addresses the provision of feedback (“I identify mistakes in the conduction of hand hygiene in other persons”), one item addresses feedback reception (“Depending on the situation, I accept feedback if others correct me for an error in hand hygiene”), and one item addresses the speaking up behavior after incorrect hand disinfection (“I correct others readily if I perceive a mistake in hand hygiene”).

*Assessment of fellow students, qualified nurses, and physicians*. We used a 5-item instrument for the assessments of fellow students (α = 0.78, ω = 0.81) and a three-item-instrument for each of the three professional groups (physicians: α = 0.70, ω = 0.70 and nurses: α = 0.72, ω = 0.77). The scales included the same aspects as the self-assessment: the situational application of knowledge in hygienic hand disinfection (e.g., “My colleagues conduct hand hygiene if indicated in a situation”), feedback reception (“Depending on the situation, nurses accept feedback if others correct them for an error in hand hygiene”), and speaking up after incorrect hand disinfection (“Physicians correct others readily if they perceive a mistake in hand hygiene“). However, the provision of feedback was not included.

*ICAP Model.* The frequency of applying passive, active, creative, and interactive teaching formats in IPC courses was measured on an ascending 5-point Likert scale from one (never) to five (always) using an eight-item instrument: one item assessed the passive format (“I am provided with learning objectives prior to the lessons”)”, two items assessed the creative format (e.g., “I am presenting prepared issues that is done alone or in groups” α = 0.72) two items assessed the active format (e.g., “I am involved in the learning lesson”, α = 0.68) and three items assessed interactive teaching formats (e.g., “I am working in groups together with other students”, α = 0.61, ω = 0.71). The disparity between alpha and ω suggests that a non-normality distribution of the scale might have led to the low Cronbach’s alpha, given that McDonald’s omega is more robust and accurate across all distributions. It should be noted, that McDonald’s omega could not be assessed in cases where there are fewer than three items in the scale.

*Feedback and Metacognition.* We measured the manifestation of teachers’ feedback behavior regarding students’ improvement in hand hygiene on an ascending 5-point Likert scale, with one item focusing on educational status and progress (“I am receiving feedback concerning my learning status and progress”). We used the next item to measure the aspects of metacognition by asking the students to report on the extent their teachers stimulate them to reflect on their current stage of learning and the improvement they have made (“I am getting support concerning my ability to reflect on my learning status and progress”).

*Self-reported hand hygiene compliance* was assessed in 12 questions. The participants were asked to specify how often they perform hand disinfection in nine indicated moments typically encountered in daily routine (one World Health Organisation (WHO) indication 3, two WHO 1, 2, 4, and 5 respectively) in accordance with the WHO Hand Hygiene guidelines [1, 33]: “In the following situations I conduct hygienic hand disinfection”:

1. After moving a used patient bed (WHO 5).
2. After contaminating your hand with urine (WHO 3).
3. Before connecting an infusion to an IV line (WHO 2).
4. After shaking hands with a patient (WHO 4).
5. Before connecting a urinary catheter to a collection bag (WHO 2).
6. After helping a patient up after a fall (WHO 4).
7. Before shaking hands with a patient (WHO 1).
8. Before positioning a patient on an operation table (WHO 1).
9. After picking up a patient’s towel that has fallen to the ground in the lavatory (WHO 5).

Additionally, we added three distractor situations not explicitly indicated by the 5 WHO Hand Hygiene guidelines (e.g., “after preparing sterile IV medication”). Hence, these items tested factual and situational knowledge and the self-assessment. Higher ratings pointed to higher occurrence: (1) never (< 1%), (2) seldom (1–25%), (3) sometimes (25–75%), (4) commonly (75–99%), and (5) always (> 99%). In addition, we computed separate index scores for indicated situations (α = 0.81, ω = 0.81) and non-indicated situations (α = 0.71, ω = 0.76).

Patient safety was assessed using two separate items to measure the self-estimated degree of patient safety. The substantial harm to patients was estimated as: “The credible maximum effect of omitted hand hygiene is insignificant – (1) without consequence, (2) minor – without any long-lasting effect, (3) severe – with longer hospital stay), (4) critical – with long-lasting effect, and (5) lethal “. Furthermore, we tested the estimation of the occurrence of these events: “How often is a patient harmed in your educational environment? (1) Uncommon (once > 3 years), (2) seldom (once every 3 years), (3) moderate (once a year), (4) often (once every 3 months), (5) very often (once a month)". The items are based on the ISO 31000 scales in risk management.

### Statistical methods

Data analysis was conducted by IBM SPSS Software Version 29 (IBM SPSS Statistics for Windows, Armonk, IBM, New York, NY, USA). After inspecting reliability indices for ICAP Subscales and for OCE dimensions, we tested the first set of hypotheses using one-way ANOVA with bootstrapping (Biased-corrected and accelerated (BCa), based on 1,000 bootstrap samples). Using two-step cluster analysis, we tested the second set of hypotheses. We included the self-assessment of competencies and the instrument measuring the self-reported hand hygiene protocol adherence, i.e., the frequency of hand disinfection in indicated situations following the WHO guidelines. The discrepancies among the identified clusters between self-assessment, self-reported frequency of indicated as well not indicated situations were compared. The clusters with high self-assessment and poor self-reported compliance in indicated situations were considered as overestimated. The third hypothesis was tested using the macro “Process” by Hayes, 2017 (model 1) [34]. The items belonging to one of the ICAP dimensions served as a focal predictor and overplacement (difference between own competencies and the competencies of fellow students) served as the criterion variable. Feedback and metacognition were each considered as moderator variables in the separate regression equation. For testing moderation effects, we applied the BCa Method based on 1,000 bootstrap samples for our calculation of moderation effects via multiple linear regression. Additionally, to obtain data points for visualizing the effects, we employed the previously mentioned Model 1 of the “Process” macro, which employs bias-corrected bootstrapping based on 5,000 bootstrap samples. Furthermore, we mean-centered the variables to avoid multicollinearity, especially between the predictor, moderator, and interaction term, which is a common problem in the moderation analysis.

## Results

### Demographics

A total of 94 “completers” of 196 responders were included in the study. The sample comprised 17 (18.06%) male and 74 (78.72%) female participants. Three missing values were identified in the dataset for this variable. The sex ratio (male to female) of the sample was very close to that of the population of HCPs and nursing students in Germany [35]. Of the 94 completers, 11 did not want to state their age. From the 83 participants, the youngest was 17 years, and the oldest was 46 years of age. The median age was 22 years with first and second quartile being 20 and 24 respectively. It is important to note that all participants, including those who chose not to state their age, were required to be over 16 years of age, since they would not receive the invitation link otherwise. Of 94 participants, 40 were OTA, 11 were ATA and 43 were GKP students. Seventeen participants reported that they had successfully completed another professional training which qualified them to work in the medical sector before beginning their present studies. Table 1 describes the sample characteristics with median, first and third quartile (Q1, Q3) for continuous and *n* (%) for categorical variables.

**Table 1:** Demographic characteristics of the sample

Characteristic		*N* (%)	Median	Q1, Q3
Age^a^		83 (88.29%)	22	20, 24
Gender^a^	Male	17 (18.06%)		
	Female	74 (78.72%)		
Students	OTA (“Operationstechnische:r Assistent:in”; surgical assistent)	40 (43.00%)		
	ATA (“Anästhesietechnische:r Assistent:in”; anesthesia technical assistent)	11 (11.70%)		
	General nursing students	43 (45.74%)		
Previously completed medical training	Yes	17 (18.09%)		
	No	77 (81.91%)		

*Note*. Q1: first (25%) quartile, Q2: second (75%) quartile. ^a^11 missing values stored for the variable age and three missing values stored for the variable gender

### Hand hygiene teaching methods

Within the questionnaire, trainees reported on the methods used to teach hand hygiene. Frontal teaching was cited by 84%, while simulation training, despite available Skills Labs (80%), was accessed by only 52%. Practical exercises were conducted by 86% of trainees, while 32% engaged in case studies, and 43% had reflective discussions. Responses largely matched perceptions of teaching methods occurrence, indicating realistic assessments. The results accordingly indicate that frontal teaching still holds significant importance. However, since only the trainees’ perspective was recorded, a comprehensive assessment of the teaching methods could not be carried out.

### Hypothesis H1

Nursing students (*M*_*1*_ = 4.18, *SD* = 0.48) rated themselves to be significantly better than:

1. Fellow students (M_2_ = 3.81, *SD* = 0.66); (*t*(93) = 5.72; *p* < 0.001; BCa 95% [0.37,0.81]; Cohen’s D_z_ = 0.62).
2. Nurses (*M*_*3*_ = 3.10, *SD* = 0.79); (*t*(93) = 12.96; *p* < 0.001; BCa 95% [0 0.94,1.23]; Cohen’s D_z_ = 0.82)
3. Physicians (*M*_*4*_ = 2.43, *SD* = 0.81); *t*(93) = 18.73; *p* < 0.001; BCa 95% [1.58,1.92]; Cohen’s D_z_ = 0.91) regarding hand hygiene competencies.

One-way ANOVA revealed that the extent of overplacement effects did not differ between the students in different schools and training years (*F*(2,91) = 0.75; *p* = 0.93.) Similarly, the intensity of overplacement effects did not differ between the students in different educational courses: OTA, GKP and ATA (*F*(2,91) = 1.57; *p* = 0.12).

### Hypothesis H2

We performed a two-step cluster analysis to test the hypothesis H2. The risk estimation item was dichotomized as follows: the answers “without consequence”, “minor – without any long-lasting effect”, “severe-with longer hospital stay”, an “critical – with long-lasting effect” was assigned the value of (1) In addition, the alternative “lethal” was assigned the value of (2). Therefore, the dichotomous item was included as a categorical variable in the cluster analysis. In contrast, self-assessment of own competencies, self-reported hand hygiene compliance in indicated situations, and self-reported hand hygiene compliance in situations not requiring hand hygiene according to the WHO were included as continuous variables in the two-step cluster analysis. We obtained a three-cluster solution with a mean silhouette coefficient > 0.3, indicating a good quality of clustering.

Cluster A (*n* = 29): Members of the cluster assessed their competencies lower than the members of the other two clusters (*M* = 3.94). Correspondently, their self-reported hand hygiene compliance was lower than in the two other clusters in both indicated (*M* = 3.81) and not indicated (*M* = 3.50) situations. All participants in this cluster reported that the maximum harm for patients after omitted or incorrect hand disinfection cannot be lethal.

Cluster B (*n* = 36): Members of the largest cluster assessed their competencies in hand hygiene highly (*M* = 4.35). Their self-reported hand hygiene compliance was also higher than the other two clusters *(M* = 4.67 for indicated and *M* = 4.55 for not indicated hand hygiene according to the WHO protocols). The members of this cluster also estimated the maximum risk for patients when the WHO protocol was not followed as not lethal.

Cluster C (*n* = 27): The members of this group estimated their hand hygiene competencies relatively highly *(M* = 4.25). Correspondingly, the hand hygiene compliance in both indicated *(M* = 4.52) and not indicated *(M* = 4.37) scenarios was also high. The participants in this cluster accurately assessed the maximum risk for patients after inadequate hand hygiene procedures as lethal.

### Hypothesis H3

**Fig. 1:**
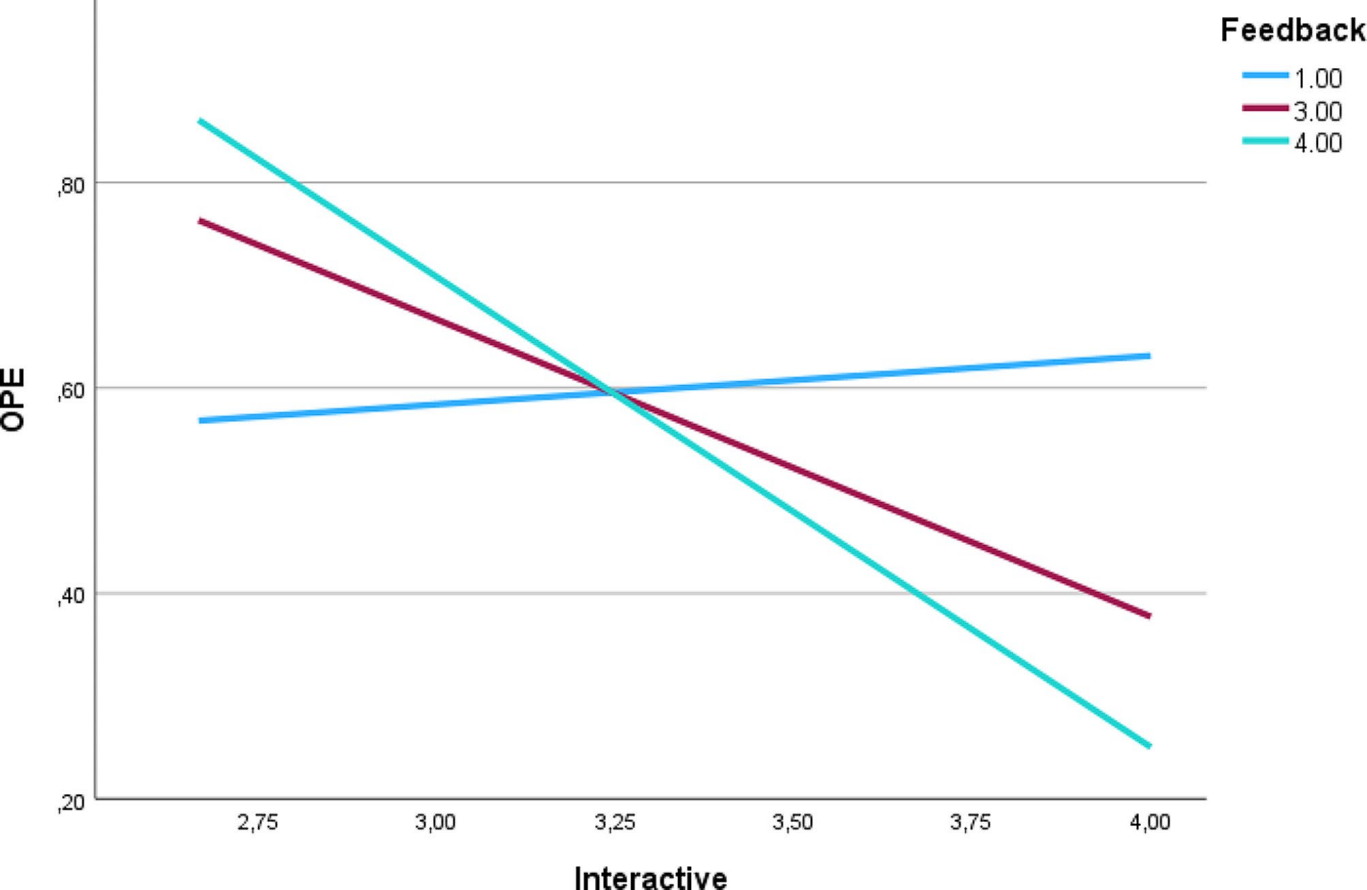
Simple slope analysis of the effects of interactive teaching format (Interactive) on overplacement (OPE) moderated by feedback provision based on the self-reported data of nursing students. Y-axis shows overplacement while x-axis shows the use of frequent interactive learning by trainers and educators in hand hygiene, ranging from one (completely disagree) to five (completely agree). The three graphs show the effects for seldom (blue), medium (red) and frequent feedback (green): When feedback is used seldomly, interactive formats have no statistical significant effect on overplacement, i.e., with more frequent reception of feedback, overplacement can be significantly reduced

We performed moderation analyses using multiple linear regressions and the Model 1 of macro “Process” for SPSS. The first set of analyses was performed with passive, active, constructive, and interactive teaching formats as predictors and overplacement as a criterion variable. Feedback was considered a moderator variable influencing the relationship between the predictor and the criterion variable. The interaction term was not significant (*p* > 0.05) in all analyses except in the analysis of the effects of interactive teaching formats on overplacement moderated by metacognition. Consequently, increased feedback resulted in decreased overplacement in the interactive learning format. The results of the analyses with feedback as a moderator are displayed in supplement B Tables 1, 2, 3 and 4. Simple slope analyses of the interactive learning format, feedback and overplacement are shown in Fig. 1.

**Fig. 2:**
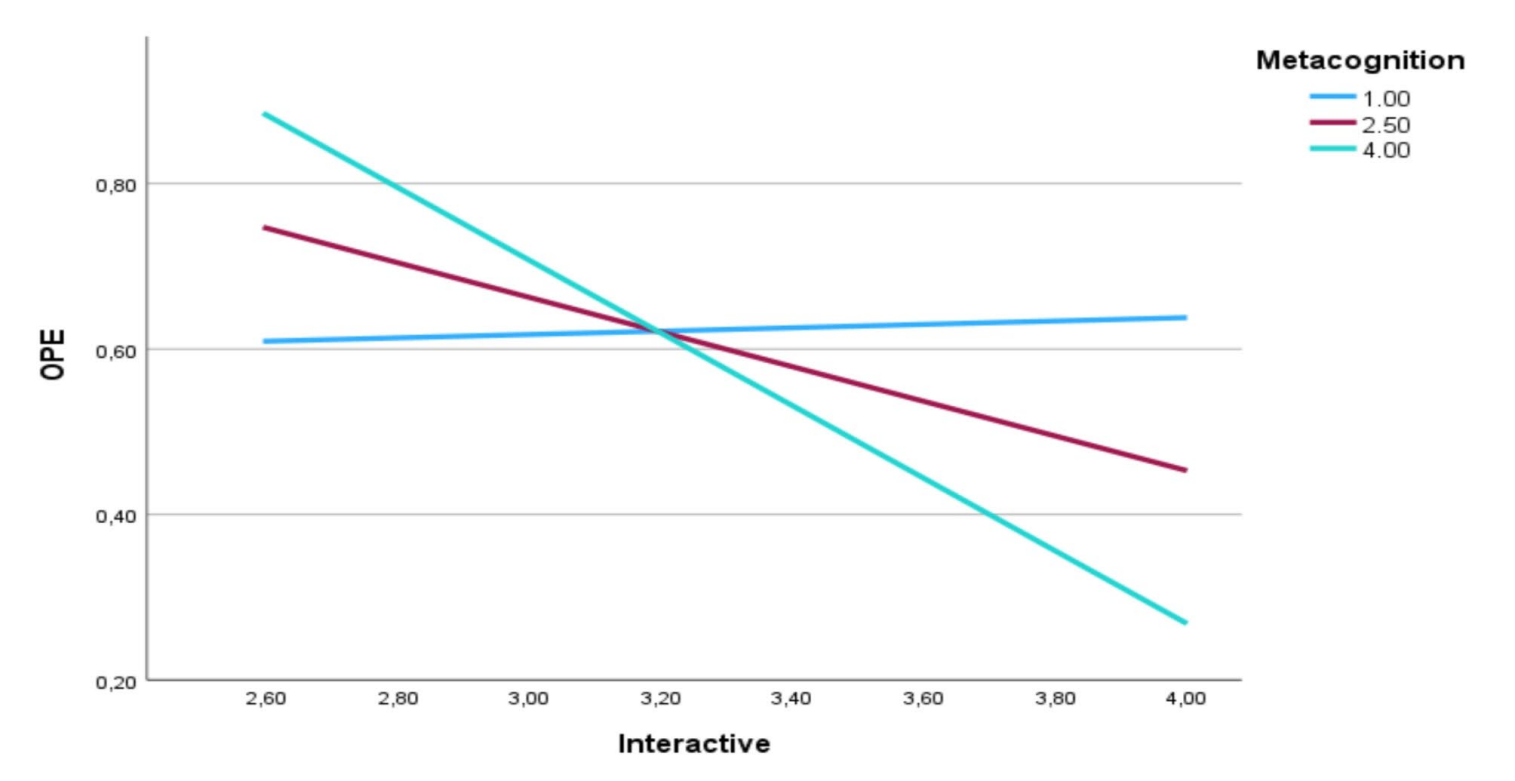
Simple slope analysis of the effects of interactive teaching format (Interactive) on overplacement (OPE) moderated by metacognition based on the self-reported data of the nursing students. In this analysis learning formats were predictor variables, metacognition a moderator and overplacement a criterion variable. Y-axis shows overplacement while x-axis shows the use of frequent interactive learning format in the classes (one “strongly disagree” to five “completely agree”). The three graphs show the effects for seldom (blue), medium (red) and frequent induction of metacognition (green) by trainers and educators in hand hygiene: The effect of interactive learning on overplacement was moderated by metacognition. The negative effect of interactive teaching format on overplacement was significant when the value of induced metacognition was high, i.e., interactive format can reduce overplacement only by promotion of self-reflective learning and metacognition

The second set of moderation analyses was performed with four different teaching formats (passive, active, constructive, and interactive) as predictor variables, metacognition as moderator and overplacement as a criterion variable. Thus, the effect of the interactive teaching format on overplacement was moderated by metacognition. Moreover, the negative effect of interactive teaching format on overplacement was significant when the value of metacognition was high. In other words, the interactive format can significantly reduce overplacement only when the promotion of self-reflective learning and metacognition is strong. The effects of passive, active, and constructive formats on overplacement were not significant and were not moderated by metacognition. Supplement B Tables 5, 6, 7 and 8 show the results of the regression-based moderation analyses. Simple slope analyses of interactive learning format, metacognition, and overplacement are shown in Fig. 2.

## Discussion

### Key results

To our knowledge, this is the first study examining the correlation between OCE on hand hygiene in nursing education. The following key results emerge from our study:

Overplacement effects regarding hand hygiene can be significantly detected in nursing students compared to fellow students, supervisors, and nurses with high effect sizes for each group, thereby confirming hypothesis one. This aligns with findings of prior studies on hand hygiene in postgraduates [6, 8–10], in convenience sampling [7], and medical students [27]. However, when considering the different groups of nursing students, no differences between the groups could be identified, and there was no correlation of the study year towards OCE, mirroring findings among medical students [27].

Second, we demonstrated overestimation in a large proportion of the students – partially confirming hypothesis two. We detected three types of respondents that can be compared to the three clusters observed in postgraduates in a parallel study conducted and published in 2022 [8]: cluster A exhibited low overplacement and overestimation and struggled with correct risk stratification in case of omitted hand hygiene. Cluster B showed high self-estimation but also failed to accurately assess the maximum risk of omitted hand hygiene. These two clusters corresponded to the “recruitables” (overconfident but motivated to learn) or “unawares” (overconfident and not motivated to learn) clusters in the previous study. However, as we did not assess learning motivation [36–38] in this study, this differentiation is not possible, highlighting opportunities for future research to integrate methodologies of this examination and the parallel one [8]. Cluster C demonstrated characteristics similar to “experts” in the other study, displaying justifiable confidence and motivation. Nevertheless, we cannot definitively label Cluster C as “experts” since learning motivation was not measured, and some errors in hand hygiene indications were observed. However, cluster C is justifiably confident as they correctly assessed the risk of omitted hand hygiene.

Third, we hypothesized that the stimulation of self-reflective learning by providing feedback, a prerequisite of metacognition, would reduce OCE. We found that the interactive teaching format, coupled with relevant feedback and stimulation of metacognitive reflection, was effective in reducing the effect in the interactive teaching format. The literature supports this finding, demonstrating that feedback and metacognition helps calibrate learners [39]. Unfortunately, the effect of high overplacement in comparison to low overplacement in hand hygiene remains unclear, whereas Lamping et al. could show an inverse correlation between overestimation and hand hygiene compliance [9] – but not for overplacement (better-than-average effect).

To overcome OCE and foster the calibration of one’s own capabilities, learners depend on their teachers and educators whether they choose interactive learning formats and thereby promote metacognition. In other words, interactive learning in infection control without the promotion of metacognition fails to alleviate OCE. As evident from the results of this study, traditional frontal education remained the predominant teaching format advocated by educators. Therefore, teachers and educators themselves need robust competencies in education as they not only have to facilitate interactive learning sessions but also need to encourage self-reflection and self-assessment. It is important to consider how educators are trained in infection control, as research suggests they may also exhibit OCE. This trend extends to various medical skills, such as basic life support, emphasizing the need for ongoing calibration and oversight to maintain teaching effectiveness [40, 41].

Hence, by choosing interactive learning with encouraging metacognitive reflection, teachers will have a greater opportunity to prevent imparting incorrect information and generating false knowledge. Moreover, this approach would result in more effective teaching, avoiding the waste of valuable educational time and resources.

### Limitations

Our study faces several limitations. The low response rate [42] renders it susceptible to selection bias and it had also a small sample size [43]. Additionally, the study was conducted solely in Germany, making it difficult to generalize the findings to other countries and cultures. Intercultural differences are known for both overplacement and overestimation, although OCE are innate to human beings. Nevertheless, our findings synchronized with parallel and preceding studies in the field of psychological factors in infection control education, laying the groundwork for future examinations of their methodologies.

A further limitation is that we assessed overestimation by self-estimations, which could be flawed in hand hygiene [9], and we were only able to assess a part of the learning dimensions [44]. Consequently, future studies should comprise more objective assessments across all learning dimensions, such as direct observations of practical skills, which is far more resource- and time-consuming. However, our results may guide future investigations into the effective planning of such investigations. Finally, due to the cross-sectional design of the study, no causal interpretations are feasible.

### Interpretation

Despite the limitations, this study successfully demonstrated that OCE, especially overplacement, in hand hygiene are prevalent across all educational stages, consistent with previous research. Furthermore, it revealed a distinction between knowledge of when to perform hand hygiene and understanding the quality of the practice. To address this, we utilized two distinct self-assessment instruments in our study. One focused on proficiency and qualitative aspects, while the other centered on self-reported adherence quantity in real-life situations. This approach enabled us to comprehensively capture both the quantity and quality of students’ hand hygiene practices and perceptions. We observed that self-assessment of hand hygiene competencies’ quality does not differ significantly from self-reported adherence in real-life scenarios.

Furthermore, we could show that the overplacement effect could be reduced by interactive education and stimulating metacognition. In other teaching formats, feedback had no effect on overplacement. However, the impact of overplacement in infection control and its relationship with the development of infections remains unknown. Nevertheless, it is known to inhibit self-assessment and metacognition, which could potentially compromise patient safety due to inadequate education in hand hygiene.

Concerning students, our findings suggest the potential for a Matthews effect [45]: Students with metacognition may improve their competence and confidence when provided with feedback in interactive learning formats. Conversely, students with limited metacognitive capabilities or fewer opportunities for development may struggle to progress in their competencies. This effect could be similarly demonstrated in a prior study by Caris et al. in the context of safety culture [46].

Our study revealed that individuals displaying OCE, as observed in Cluster A and B, exhibited flawed risk assessments regarding omitted or incorrect hand hygiene, aligning with findings from a similar study [8]. Previous research has indicated that a higher risk assessment serves as a motivation for learning, emphasizing the importance of evaluating this aspect in future projects [8, 47].

Moreover, there is a critical need for highly educated instructors capable of providing effective feedback and conducting interactive training in infection control. Consequently, integrating didactic strategies into the curricula of these educators is essential.

### Implication for practice and future studies

The implications of our findings for practice highlight the importance of incorporating interactive teaching formats that promote metacognitive reflection in IPC training. Educators should aim to stimulate accurate self-assessment and self-reflection among students to reduce OCE, particularly in hand hygiene education. Additionally, our study emphasizes the need for well-educated teachers who possess robust competencies in education, including the ability to provide effective feedback and facilitate interactive learning sessions.

For future studies, there is a call to address the limitations identified in our research by conducting more objective assessments across all learning dimensions. Direct observations of practical skills could provide important insights into students’ actual hand hygiene practices and perceptions. Exploring the potential relationship between the Hawthorne effect (the phenomenon where individuals modify their behavior in response to being observed or participating in a study, typically resulting in improved performance or behavior [48]) and OCE in hand hygiene education can be one of the future key points for understanding how observation impacts students’ self-assessment of their hand hygiene practices. Insights gained might enhance training strategies, promote accurate self-assessment, and reduce OCE among students. Furthermore, exploring the impact of overplacement on IPC and the development of infections remains an important area for investigation. Future research should also evaluate the effectiveness of different teaching formats and interventions in reducing OCE and enhancing patient safety in healthcare settings.

### Electronic supplementary material

Below is the link to the electronic supplementary material.

Supplementary Material 1

Supplementary Material 2

## Data Availability

The datasets used and analysed during the current study are available from the corresponding author on reasonable request.

## Abbreviations

HCP: health care professionals
OCE: overconfidence effects
ICAP: interactive, constructive, active, and passive
ATA: “Anästhesietechnische: r Assistent: in” (anesthesia technical assistant)
OTA: “Operationstechnischer: r Assistent: in ” (surgical assistant)
IPC: infection prevention and control
WHO: World Health Organisation
IV: Intravenous
α: Cronbach’s alpha
ω: McDonald’s Omega
BCa: Bias-corrected and accelerated
ANOVA: Analysis of variance
