# Supplementary material for: Interaction between overconfidence effects and training formats in nurses’ education in hand hygiene
Source: BMC Nurs. 2024 Jul 2;23:451. doi: 10.1186/s12912-024-02020-w (PMC11218338; doi:10.1186/s12912-024-02020-w)
Supplement: Supplementary file 1 — Supplementary Material 1 [file 12912_2024_2020_MOESM1_ESM.docx]

SUPPLEMENT A

Translation of the German questionnaire by the authors (DeepL Translation and manual idiomatic verification by the authors.

**Survey on infection prevention and patient safety**

Dear trainees,

We invite you to participate in an anonymous survey on infection prevention training, which will take about 10 minutes and thank you in advance for your participation.

The background of our project is the prevention of infections acquired in hospitals. In particular, infections with multi-resistant pathogens (MRE) such as MRSA, VRE, and MRGN pose great challenges for us and you as future colleagues in the coming years, so a constant adaptation of training and later further training is necessary.

With this survey, we would like to ascertain your assessments of your own competencies in the field of infection prevention and the assessment of others:

This includes manual skills such as hand hygiene but also a corrective intervention in case of observed errors.

This voluntary and anonymous (no assessment of name or IP address) survey by the Institute for Hospital Hygiene and Infection Prevention (IKIP) and the Academy for

Health Professions Academy of the Constance District Health Association (GLKN), we want to record and use these assessments for scientific evaluation.

Our team acts independently of professional societies, market research and industry (no conflict of interest).

Your answers will be treated confidentially.

The work is carried out within the framework of the B.A. study program in Educational Science (FernUniversität in Hagen) by Ms. Julia Seidel-Fischer. The overall results are intended for publication in a

in a medical journal.

Please contact the study leaders if you have any questions about the survey or the topic.

Thank you for your participation!

With collegial greetings

Julia Seidel-Fischer and Stefan Bushuven MD

First of all, a few questions for you ...

**What year of training are you currently in?**

Pick one: 1st year of training / 2nd year of training / 3rd year of training

**What gender are you?**

Pick one: female, male, diverse/transgender/transidentical, not specified

**How old are you?**

Free text entry

**Questions about lesson planning**

**Does your school have a skills lab (teaching workshop/practice room)?**

Pick one: yes, no

**How is the topic of hand hygiene taught in your training?**

Multiple answers possible

- Frontal teaching/lecture/lecture/Simulation/skills lab/teaching workshop
- Working on case studies
- Reflective discussions with teachers and practical instructors
- Practical exercises
- Free text entry

**Which professional groups teach hygiene in your facility?:**

Multiple Answers possible

- Nursing educators
- Doctors
- Practice instructors
- Hygiene specialists
- Others

**How often does the following occur in your learning lessons on hygiene?**

Likert scale for all the following items: never, seldom, sometimes, often, always, not assessable

- I am told the learning objectives of the lesson in advance.
- I am involved in the lesson.
- I discuss the topic of hygiene with teachers and classmates.
- I work together in a group.
- I present hygiene topics I have worked on alone or in groups.
- I discuss hygiene issues with teachers and classmates, that I experience during the practical assignment.
- I practice hygienic work in a simulated environment.
- I work on given case studies and try to find a solution.

**How comprehensible are the lessons on hygiene for you?**

For all questions:

completely agree, agree, mostly agree, partly agree, agree less, do not agree at all

agree, not assessable.

- I can follow the lessons.
- I receive feedback on my learning level and progress.
- I receive guidance on reflecting on my learning and progress.
- I reflect on my learning.
- I can put what I have learned into practice in everyday life.
- I feel well informed by the lessons and prepared for the practical work.
- I am prepared for practical work.

___________________________

**Self-assessment questions**

**For all the following questions, the same Likert scale is used:**

**Strongly agree, Fairly agree, Partly agree, Somewhat agree, Strongly disagree**

- I perform hygienic hand disinfection according to the situation.
- I recognize the indications for hygienic hand disinfection.
- I select the required hand disinfectant according to the situation.
- I recognize errors in the performance of hygienic hand disinfection by other persons.
- I take corrective action when I notice a mistake in the performance of hygienic hand disinfection.
- I accept feedback appropriate to the situation when another person informs me of an error in hygienic hand disinfection.

___________________________________________________________________

**Please assess the observed behavior of your classmates!**

**For all following questions, the same Likert scale is used:**

**Strongly agree, Fairly agree, Partly agree, Somewhat agree, Strongly disagree**

- My classmates implement hygienic hand disinfection according to the situation.
- My classmates know the respective indications for hygienic hand disinfection.
- My classmates correctly select the necessary disinfectant for hygienic hand disinfection.
- My classmates correct me according to the situation when they notice a mistake in my hand disinfection.
- My classmates accept feedback and advice appropriate to the situation when they are asked about a hygiene error.

___________________________________________________________________

**Please assess the observed behavior of postgraduate nurses!**

**For all the following questions the same Likert scale is used:**

**Strongly agree, Fairly agree, Partly agree, Somewhat agree, Strongly disagree**

- Nurses implement hygienic hand disinfection according to the situation.
- Nurses know the respective indications for hygienic hand disinfection.
- Nurses correctly select the necessary disinfectant for hygienic hand disinfection.
- Nurses correct me according to the situation when they notice a mistake in my hand disinfection.
- Nurses accept feedback and advice appropriate to the situation when they are asked about a hygiene error.

___________________________________________________________________

**Please assess the observed behavior of postgraduate physicians!**

**For all following questions, the same Likert scale is used:**

**Strongly agree, Fairly agree, Partly agree, Somewhat agree, Strongly disagree**

- Physicians implement hygienic hand disinfection according to the situation.
- Physicians know the respective indications for hygienic hand disinfection.
- Physicians correctly select the necessary disinfectant for hygienic hand disinfection.
- Physicians correct me according to the situation when they notice a mistake in my hand disinfection.
- Physicians accept feedback and advice appropriate to the situation when they are asked about a hygiene error.

**How do you assess the risk of omitted hand disinfection?**

**The credible maximum harm of omitted hand disinfection is**

- Insignificant
- minor - but no lasting damage
- noticeable - with prolonged stay in hospital
- critical - with permanent physical damage
- catastrophic - resulting in death
- not specified
- do not know

In your training environment, how often does a patient suffer the estimated harm due to a failure to disinfect hands?

- less frequently than once in 3 years
- more often than once in 3 years
- more often than once a year
- more often than once in 3 months
- more often than once a month
- not specified
- I do not know

**How would you rate your own behavior in everyday medical practice?**

For all questions:

The percentages refer to your estimate of how often you carry this out in corresponding cases in percentages.

I implement hygienic hand disinfection in the following situations:

- Before positioning a patient in bed or on an operating table
- After saying goodbye to a patient with a handshake
- Before entering a patient's room during a ward round
- After accidentally contaminating one's own hand with a patient’s urine.
- Before connecting an IV line to an indwelling peripheral venous cannula
- After a meal during the lunch break
- Before greeting a patient with a handshake
- After helping up a fallen patient
- After picking up a dropped towel from a patient in the bathroom.
- After putting down a used patient's bed in an examination room.
- Before attaching a new urine bag to a urinary bladder catheter.
- After preparing a medication

**Speaking Up**

Likert scale applying to all following answers:

Strongly agree, Fairly agree, Partly agree, Somewhat agree, Strongly disagree, No answer

**If I notice a mistake in the hygienic hand disinfection of other persons, I intervene to correct it at**

- Visitors of patients
- Cleaning staff
- Nursing trainees
- Registered nurses
- Practice supervisors
- Assistant doctors
- Senior physicians
- Head physicians

Please rate:

**In which training year should training in hygienic hand disinfection start?**

Please select: 1,2 or 3

**How often should the training in hygienic hand disinfection be repeated in the sense of a "refresher" during the training?**

**Please rank the following patient safety training contents in order of importance! (1= most important)**

- Safety in the use of medication (Medication Safety)
- Measures of infection prevention (hospital hygiene)
- User safety in diagnostics (clinical picture, laboratory parameters, imaging)
- Strategies in surveillance and therapy of sepsis (blood poisoning)
- Strategies on cyber security of medical products and data protection
- Strategies to prevent unnecessary hospital admissions

**Please rank the contents in hospital hygiene and infection prevention in order of importance for training! (1= most important)**

- Handling personal protective equipment (gloves, gowns, face masks, ...)
- Hygienic hand disinfection
- Dealing with isolation measures (for patients with multi-resistant pathogens such as MRSA)
- Surface disinfection
- Medical device reprocessing (e.g., surgical instruments)
- Behavior in the event of an outbreak (accumulation of infectious diseases)
- Dealing with pre-analytics ("How do I collect, store and send laboratory samples?")
- Diagnostics of infectious diseases (e.g., clinical examination, interpretation of laboratory results, imaging)
- Therapy of infectious diseases (e.g., use of antibiotics)

**Suggestions for improvement, suggestions, and comments**

Do you have any suggestions for improvement, special experiences, or comments on infection prevention? Let us know!

Freetext entry

**Done! Would you like to know more? Do you have any questions or uncertainties?**
