# Supplementary material for: Interaction between overconfidence effects and training formats in nurses’ education in hand hygiene
Source: BMC Nurs. 2024 Jul 2;23:451. doi: 10.1186/s12912-024-02020-w (PMC11218338; doi:10.1186/s12912-024-02020-w)
Supplement: Supplementary file 2 — Supplementary Material 2 [file 12912_2024_2020_MOESM2_ESM.docx]

SUPPLEMENT B

**Supplement B Table 1.** Results of moderation analysis of feedback in the effect of passive teaching format on overplacement

| **Predictor** | **β** | **β BCa 95% CI lower** | **β BCa 95% CI upper** |
| --- | --- | --- | --- |
| Passive teaching format | 0.00 | -0.29 | 0.29 |
| Feedback | 0.17 | -0.15 | 0.49 |
| Interaction  Passive teaching format *  Feedback | -0.04 | -0.14 | 0.06 |

Criterion variable is overplacement.

**Supplement B Table 2.** Results of moderation analysis of feedback in the effect of active teaching format on overplacement

| **Predictor** | **β** | **β BCa 95% CI lower** | **β BCa 95% CI upper** |
| --- | --- | --- | --- |
| Active teaching format | 0.07 | -0.28 | 0.42 |
| Feedback | 0.29 | -0.07 | 0.65 |
| Interaction  Active teaching format *  Feedback | -0.08 | -0.19 | 0.03 |

Criterion variable is overplacement.

**Supplement B Table 3**. Results of moderation analysis of feedback in the effect of constructive teaching format on overplacement

| Predictor | β | β BCa 95% CI lower | β BCa 95% CI upper |
| --- | --- | --- | --- |
| Constructive teaching format | 0.00 | -0.28 | 0.27 |
| Feedback | 0.17 | -0.06 | 0.40 |
| Interaction  Constructive teaching format *  Feedback | -0.06 | -0.15 | 0.03 |

Criterion variable is overplacement.

**Supplement B Table 4.** Results of moderation analysis of feedback in the effect of interactive teaching format on overplacement

| Predictor | β | β BCa 95% CI lower | β BCa 95% CI upper |
| --- | --- | --- | --- |
| Interactive teaching format | 0.22 | -0.12 | 0.55 |
| Feedback | 0.55 | 0.19 | 0.90 |
| Interaction  Interactive teaching format *  Feedback | -0.17 | -0.28 | -0.06 |

Criterion variable is overplacement.

**Supplement B Table 5.** Results of moderation analysis of metacognition in the effect of passive teaching format on overplacement

| **Predictor** | **β** | **β BCa 95% CI lower** | **β BCa 95% CI upper** |
| --- | --- | --- | --- |
| Passive teaching format | 0.01 | -0.29 | 0.32 |
| Metacognition | 0.19 | -0.17 | 0.54 |
| Interaction  Passive teaching format *  Metacognition | -0.05 | -0.16 | 0.06 |

Criterion variable is overplacement.

**Supplement B Table 6.** Results of moderation analysis of metacognition in the effect of active teaching format on overplacement

| **Predictor** | **β** | **β BCa 95% CI lower** | **β BCa 95% CI upper** |
| --- | --- | --- | --- |
| Active teaching format | 0.21 | -0.16 | 0.58 |
| Metacognition | 0.40 | 0.02 | 0.78 |
| Interaction  Active teaching format *  Metacognition | -0.13 | -0.26 | 0.00 |

Criterion variable is overplacement.

**Supplement B Table 7.** Results of moderation analysis of metacognition in the effect of constructive teaching format on overplacement

| **Predictor** | **β** | **β BCa 95% CI lower** | **β BCa 95% CI upper** |
| --- | --- | --- | --- |
| Constructive teaching format | 0.04 | -0.26 | 0.33 |
| Metacognition | 0.55 | -0.05 | 0.48 |
| Interaction  Constructive teaching format *  Metacognition | -0.17 | -0.19 | 0.02 |

Criterion variable is overplacement.

**Supplement B Table 8.** Results of moderation analysis of metacognition in the effect of interactive teaching format on overplacement

| **Predictor** | **β** | **β BCa 95% CI lower** | **β BCa 95% CI upper** |
| --- | --- | --- | --- |
| Interactive teaching format | 0.17 | -0.19 | 0.54 |
| Metacognition | 0.49 | 0.08 | 0.90 |
| Interaction  Interactive teaching format *  Metacognition | -0.15 | -0.29 | -0.02 |

Criterion variable is overplacement.
